# Efficacy of a plant‐produced virus‐like particle vaccine in chickens challenged with Influenza A H6N2 virus

**DOI:** 10.1111/pbi.13219

**Published:** 2019-08-22

**Authors:** Tanja Smith, Martha M. O'Kennedy, Daniel B.R. Wandrag, Modupeore Adeyemi, Celia Abolnik

**Affiliations:** ^1^ Department of Production Animal Studies Faculty of Veterinary Science University of Pretoria Pretoria South Africa; ^2^ Biosciences Council for Scientific and Industrial Research Pretoria South Africa

**Keywords:** avian influenza, *Nicotiana benthamiana*, VLP vaccine, H6N2, chickens, viral shedding

## Abstract

The efficacy, safety, speed, scalability and cost‐effectiveness of producing hemagglutinin‐based virus‐like particle (VLP) vaccines in plants are well‐established for human influenza, but untested for the massive poultry influenza vaccine market that remains dominated by traditional egg‐grown oil‐emulsion whole inactivated virus vaccines. For optimal efficacy, a vaccine should be closely antigenically matched to the field strain, requiring that influenza A vaccines be updated regularly. In this study, an H6 subtype VLP transiently expressed in *Nicotiana benthamiana* was formulated into a vaccine and evaluated for efficacy in chickens against challenge with a heterologous H6N2 virus. A single dose of the plant‐produced H6 VLP vaccine elicited an immune response comparable to two doses of a commercial inactivated H6N2 vaccine, with mean hemagglutination inhibition titres of 9.3 log_2_ and 8.8 log_2_, respectively. Compared to the non‐vaccinated control, the H6 VLP vaccine significantly reduced the proportion of shedders and the magnitude of viral shedding by >100‐fold in the oropharynx and >6‐fold in the cloaca, and shortened oropharyngeal viral shedding by at least a week. Despite its potency, the cost of the antigenic mismatch between the inactivated H6N2 vaccine and challenge strain was evident not only in this vaccine's failure to reduce viral shedding compared to the non‐vaccinated group, but its apparent exacerbation of oropharyngeal viral shedding until 21 days post‐challenge. We estimate that a kilogram of plant leaf material can produce H6 VLP vaccines sufficient for between 5000 and 30 000 chickens, depending on the effective dose and whether one or two immunizations are administered.

## Introduction

Influenza A virus serotypes are designated by the combination of two major surface antigens, hemagglutinin (HA; types H1 to H16) and neuraminidase (types N1 to N9), that elicit protective humoral responses in the host (OIE, 2018). Avian influenza (AI), a fast‐spreading and lethal disease of poultry with zoonotic potential, is usually caused by highly pathogenic (HPAI) viruses of the H5Nx or H7Nx subtypes (where x denotes any of the nine neuraminidase subtypes), and the detection of both HPAI and their low pathogenic (LPAI) precursor viruses is notifiable to the World Organization for Animal Health (OIE, 2018). Subtype H6Nx viruses are classified as LPAI and are therefore non‐notifiable yet countries such as Taiwan, the United States of America and South Africa have been forced to apply official control measures during persistent H6Nx outbreaks (Lee *et al*., 2016; Rauff *et al*., 2016; Woolcock *et al*., 2003). H6Nx is one of the few LPAI subtypes with a genetic ability to form stable lineages in poultry. Infection typically causes increased mortalities, drops in egg production and respiratory disease with increased secondary bacterial infections requiring antibiotic treatment (Kinde *et al*., 2003; Rauff *et al*., 2016; Woolcock *et al*., 2003). More concerning, emerging poultry‐origin H6Nx viruses in East Asia have zoonotic potential (Ni *et al*., 2015; Wang *et al*., 2014; Wei *et al*., 2013; Xin *et al*., 2015).

Vaccination against AI, when applied properly in conjunction with rigorous monitoring and strict biosecurity measures, not only protects poultry against clinical disease but greatly reduces field virus shedding, thereby limiting further spread (Swayne *et al*., 2006). Influenza A virus, however, has a naturally high mutation rate, more so under vaccination pressure, and antigenic mismatch between field strains, and the vaccine significantly reduces the vaccine's effectiveness (Swayne and Kapczynski, 2017). For this reason, the OIE recommends that vaccine strains should be re‐evaluated every 2–3 years for efficacy against circulating field viruses and updated as needed. Since LPAI viruses do not usually produce clinical signs under experimental conditions, an efficacious vaccine should produce a statistically significant reduction in viral shedding titre and the number of birds shedding from the oropharynx or cloaca compared with a non‐vaccinated group (OIE, 2018).

The chicken‐producing industry in South Africa has been beset by sporadic H6N2 outbreaks that started in the early 2000s. At the time, an autogenous inactivated oil‐emulsion vaccine produced from a 2002 field strain by the traditional egg‐based system (AVIVAC^®^ AI, Deltamune, Pretoria, South Africa) was registered for use to protect flocks. This same vaccine is still in use 17 years later, albeit under strictly regulated conditions; no other H6 vaccines are registered in the country. The continuing outbreaks have involved two related but distinct H6N2 sub‐lineages (I and II) that derived from a common ancestor, and Rauff *et al*. (2016) provided *in silico* evidence that the commercial vaccine had accelerated the genetic and antigenic drift in the homologous sub‐lineage I field strains over an 11‐year period. The commercial vaccine continues to be used by some producers due to a perception that it provides protection against clinical disease. However, neither have clinical data been presented to prove this vaccine's efficacy, nor have any field isolates since 2002 been developed as a replacement seed strain.

The vast majority of AI vaccines licensed for use in poultry are whole inactivated virus vaccines formulated with oil emulsions to enhance immunogenicity (Swayne and Kapczynski, 2017). Due to the long production time, alternatives to traditional egg‐based influenza vaccine production have been pursued, one of which is virus‐like particles (VLPs). VLPs are self‐assembled protein structures that closely resemble the organization and conformation of native viruses but lack core genetic material, thereby producing a stable antigen that is non‐infectious. In addition to strong, long‐lasting humoral virus‐neutralizing responses, the particulate nature of VLPs, on which epitopes are displayed in dense repetitive arrays, enables them unlike subunit vaccines to induce potent T‐cell‐mediated immune responses through interaction with antigen‐presenting cells, especially dendritic cells (Bright *et al*., 2007; Quan *et al*., 2007; Shoji *et al*., 2015; Song *et al*., 2010).

Biopharming has become increasingly popular for the production of biologics, including vaccines. By making use of transient protein expression in plants, HA‐based influenza VLP candidate vaccines for humans have been produced rapidly at low cost and showed good safety and immunogenicity in pre‐clinical and clinical tests (D'Aoust *et al*., 2010; Landry *et al*., 2010). Here, a plant‐produced VLP vaccine based on the HA protein sequence of a 2016 H6N2 virus was tested for efficacy in specific‐pathogen‐free (SPF) chickens in a prime‐boost strategy, the first efficacy study for a plant‐produced VLP vaccine in an avian species. The ability of the H6 VLP vaccine to reduce viral shedding from the respiratory and gastrointestinal tracts upon challenge with a heterologous 2016 H6N2 virus was assessed in comparison with the commercial whole inactivated virus vaccine.

## Results

### VLP production, purification and confirmation of identity

Influenza VLPs containing HA (A/chicken/South Africa/N2826/2016 (H6N2)) and matrix 2 (M2) (A/New Caledonia/20/1999 (H1N1)) proteins were transiently produced in *N. benthamiana* plants lacking plant‐specific N‐glycan residues (Strasser *et al*., 2008) using agroinfiltration. The leaves of 5‐ to 8‐week plants were hand‐infiltrated with an *Agrobacterium* inoculum (AGL1; OD_600_ 1.5) containing equal parts of pEAQ‐HT+H6 and pEAQ‐HT+M2 recombinant plasmids, as the co‐expression of HA with M2 resulted in increased H6 VLP production. Six days after infiltration, the infiltrated leaves were harvested and homogenized in two volumes of buffer, followed by clarification through cheese cloth and purification using differential ultracentrifugation [20%–60% Iodixanol density gradients (OptiPrep^™^; Sigma‐Aldrich, St. Louis, MO)].

**Figure 1:**
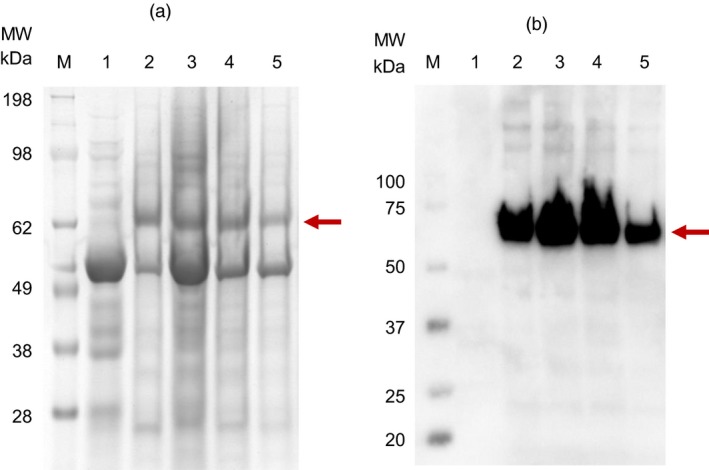
SDS‐PAGE electrophoresis (a) and Immunoblot (b) of purified plant‐produced H6 hemagglutinin. Lane 1: negative control – plant‐expressed pEAQ‐HT; lanes 2 to 4: H6 hemagglutinin present in fractions 10, 11 and 12 of the Iodixanol density gradient; lane 5: H6 hemagglutinin dialysed in 1xPBS and stabilized with trehalose. M: The SeeBlue Plus2 (a) and WesternC (b) protein molecular weight markers were used for the SDS‐PAGE and immunoblot, respectively. The arrows indicate the position of the target protein (approximately 62 kDa).

Sodium dodecylsulphate–polyacrylamide gel electrophoresis (SDS‐PAGE) and immunoblot analysis of partially purified plant extract indicated a prominent band of approximately 62 kDa (Figure 1a,b), which corresponds to the full‐length HA protein and reacted strongly with H6N2 chicken antiserum. Liquid chromatography–mass spectrometry (LC‐MS/MS)‐based peptide sequencing confirmed the 62 kDa SDS‐PAGE band to be the HA of A/chicken/South Africa/N2826/2016 (H6N2): sequence coverage of 44.1% was obtained and 35 peptides were identified with more than 95% confidence (Figure S2a). The most abundant H6 HA proteins were found to be localized in the 20%–30% Iodixanol fractions (fractions 10–12), as assessed by SDS‐PAGE and immunoblot analysis. A unique band of approximately 14 kDa was identified using SDS‐PAGE analysis in previous experiments, which was confirmed to be M2 protein using LC‐MS/MS‐based peptide sequencing: coverage of 17.5% was obtained and 5 peptides were identified with >95% confidence (Figure S2b). In early experiments, the yield of H6 HA was conservatively estimated to be 95 mg/kg leaf material using the Micro BCA™ Protein Assay Kit, accounting for approximately 20% of protein in the density gradient fractions analysed (pooled fractions 10 and 11).

**Figure 2:**
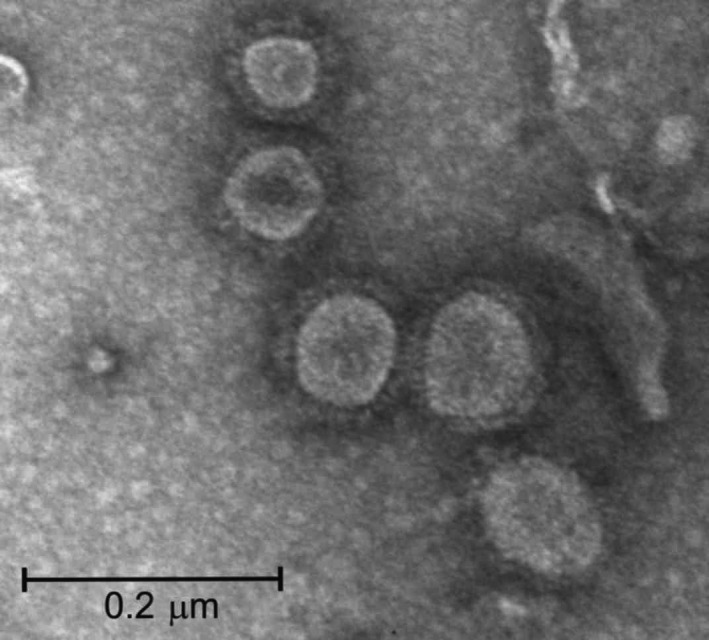
Negative stained transmission electron microscopy image of plant‐produced H6‐type influenza virus‐like particles.

The partially purified plant extract examined under the transmission electron microscopy (TEM) revealed abundant VLPs resembling native influenza viral particles (Figure 2). The VLPs were roughly spherical with particle sizes ranging in diameter from 40 to 190 nm, but most measured between 70 and 100 nm. These results were similar to a previous report for plant‐produced influenza VLPs (Lindsay *et al*., 2018). The H6 influenza VLPs were subsequently subjected to hemagglutination and hemagglutination inhibition (HI) assays to confirm functionality, yielding a titre per 25 μL of 9 log_2_ [512 HA units (HAU)] and 6 log_2_, respectively. With the HI assay, low non‐specific reactions were observed with the negative control SPF sera that were likely due to the presence of other plant proteins.

### Assessment of efficacy in chickens

#### Immune responses

To verify that the 6‐week‐old SPF chickens had no prior exposure to AI, ten chickens randomly selected prior to the start of the trial were bled. Sera were tested using IDEXX Influenza A Virus Antibody Test Kit according to manufacturer's instructions, and the sample to negative control ratio (S/N) values (0.77; 1.06; 0.80; 0.72; 0.79; 0.80; 0.75; 0.79; 0.86; 0.96) was all negative (S/N <0.5 is considered as positive). Chickens in treatment groups A [plant‐produced H6 VLP vaccine adjuvanted with Montanide^™^ ISA 71 VG (Seppic, France), 769 HAU/0.3 mL dose] and B (commercial inactivated H6N2 oil‐emulsion vaccine, EID_50_ ≥ 10^8^/0.5 mL dose) were vaccinated at 6 and 10 weeks of age and challenged 2 weeks later with a heterologous 2016 H6N2 virus (10^6^ EID_50_/0.6 mL). Group C served as the non‐vaccinated control. To assess the immune response, chickens were bled 4 weeks after the primary immunization (groups A and B), 2 weeks after booster vaccine just prior to challenge (groups A and B) and 2 weeks after viral challenge (groups A, B and C) with serological test results presented in Table 1. In addition to the antigen used for routine testing in South Africa (‘2002 HI test antigen’, which is homologous with the commercial H6N2 vaccine seed strain), the 2016 challenge virus was also included (‘2016 HI test antigen’, which is 95.77% identical to the H6 VLP's homologous strain in the HA protein). Although the most accurate quantitation of HA‐specific antibodies is obtained using a homologous or closely related virus (Swayne *et al*., 2015), the H6 VLP's homologous live virus could not be used as a test antigen due to the presence of a contaminating virulent Newcastle disease virus.

**Table 1:** Serology test results for influenza A nucleoprotein antibody ELISAs and hemagglutination inhibition (HI) assays with positive values in boldface

Treatment group	Chicken No.	10 weeks of age 4 weeks post‐primary vaccination			12 weeks of age 2 weeks post‐booster vaccination (pre‐challenge titres)			14 weeks of age 2 weeks post‐challenge		
		ELISA S/N*	H6N2 HI Log_2_ titre		ELISA S/N*	H6N2 HI Log_2_ titre		ELISA S/N*	H6N2 HI Log_2_ titre	
			2002 antigen†	2016 antigen‡		2002 antigen†	2016 antigen‡		2002 antigen†	2016 antigen‡
A: H6 VLP vaccine	A1	1.37	**4**	**8**	1.01	**8**	**10**	0.82	**5**	**7**
	A2	0.90	**7**	**10**	0.84	**10**	**11**	0.81	**7**	**9**
	A3	0.96	**6**	**9**	0.80	**7**	**9**	**0.22**	**9**	**12**
	A4	0.95	**6**	**10**	0.84	**10**	**12**	0.92	**8**	**10**
	A5	1.05	**7**	**10**	0.90	**10**	**11**	0.87	**7**	**9**
	A6	0.93	**6**	**9**	0.79	**8**	**10**	0.60	**6**	**9**
	A7	0.81	**5**	**8**	0.88	**7**	**9**	0.80	**7**	**10**
	A8	0.85	**5**	**8**	0.84	**9**	**12**	0.79	**7**	**10**
	A9	1.05	**6**	**10**	0.87	**9**	**12**	0.64	**7**	**11**
	A10	1.00	**5**	**8**	0.74	**8**	**10**	**0.43**	**9**	**12**
	A11	0.98	**7**	**12**	0.82	**8**	**11**	1.01	**6**	**8**
	A12	0.98	**9**	**10**	0.80	**9**	**11**	0.89	**9**	**12**
	GMT	0.99 ± 0.14	**6.1 ± 1.3**	**9.3 ± 1.2**	0.84 ± 0.07	**8.6 ± 1.1**	**10.7 ± 1.1**	0.73 ± 0.23	**7.3 ± 1.3**	**9.9 ± 1.6**
B: Commercial H6N2 vaccine	B1	0.90	**6**	**4**	**0.17**	**9**	**8**	**0.11**	**10**	**9**
	B2	0.64	**8**	**7**	**0.14**	**9**	**9**	**0.13**	**8**	**9**
	B3	**0.26**	**9**	**8**	**0.14**	**10**	**9**	**0.14**	**7**	**7**
	B4	**0.48**	**4**	2	**0.11**	**7**	**6**	**0.08**	**6**	**8**
	B5	**0.14**	**6**	**5**	**0.15**	**8**	**7**	**0.08**	**9**	**10**
	B6	**0.07**	**9**	**8**	**0.07**	**10**	**8**	**0.05**	**10**	**11**
	B7	**0.46**	**8**	**8**	**0.16**	**9**	**9**	**0.30**	**9**	**7**
	B8	**0.09**	**10**	**8**	**0.15**	**12**	**10**	**0.05**	**8**	**8**
	B9	**0.42**	**7**	**6**	**0.27**	**9**	**8**	**0.07**	**8**	**9**
	B10	1.07	3	2	**0.20**	**6**	**6**	**0.07**	**7**	**9**
	B11	**0.30**	**8**	**7**	**0.17**	**7**	**7**	**0.05**	**7**	**9**
	B12	**0.13**	**7**	**7**	**0.07**	**10**	**9**	**0.04**	**8**	**9**
	GMT	**0.41 ± 0.32**	**7.1 ± 2.1**	**6 ± 2.3**	**0.2 ± 0.1**	**8.8 ± 1.6**	**8 ± 1.3**	**0.1 ± 0.1**	**8.1 ± 1.2**	**8.8 ± 1.1**
C: Non‐vaccinated control	C1	nt	nt	nt	nt	nt	nt	**0.31**	**5**	**8**
	C2	nt	nt	nt	nt	nt	nt	**0.15**	**5**	**8**
	C3	nt	nt	nt	nt	nt	nt	**0.24**	3	**8**
	C4	nt	nt	nt	nt	nt	nt	**0.27**	**6**	**8**
	C5	nt	nt	nt	nt	nt	nt	**0.39**	**6**	**8**
	C6	nt	nt	nt	nt	nt	nt	**0.48**	3	**6**
	C7	nt	nt	nt	nt	nt	nt	**0.28**	3	**7**
	C8	nt	nt	nt	nt	nt	nt	**0.20**	3	**8**
	C9	nt	nt	nt	nt	nt	nt	**0.29**	**5**	**9**
	C10	nt	nt	nt	nt	nt	nt	**0.18**	**5**	**9**
	C11	nt	nt	nt	nt	nt	nt	**0.25**	3	**8**
	C12	nt	nt	nt	nt	nt	nt	**0.20**	3	**8**
	GMT							**0.27 ± 0.1**	**4.2 ± 1.3**	**7.9 ± 0.8**

*Sample to negative ratio.

†A/chicken/South Africa/W04/2002 (H6N2) antigen.

‡A/chicken/South Africa/H44954/2016 (H6N2) antigen.

GMT, geometric mean titre; nt, samples not collected for testing.

Four weeks after a single administration, chickens vaccinated with the H6 VLP vaccine (group A) had high HI titres ranging from 8 log_2_ to 12 log_2_ [geometric mean titre (GMT) of 9.3 log_2_] when tested against the 2016 H6N2 antigen, whereas the titres were markedly lower when tested against the 2002 antigen, with a GMT of 6.1 log_2_ (Table 1). Chickens vaccinated with the commercial vaccine (group B) had a greater range of H6‐specific antibody titres ranging from of 3 log_2_ (i.e. just below the positive threshold) to 10 log_2_ (GMT of 7.1 log_2_) against the homologous antigen, and when tested against the 2016 antigen, two of the chickens (B4 and B10) were HI negative, with a group GMT of only 6 log_2_. Nucleocapsid protein (NP)‐specific antibodies detected by IDEXX enzyme‐linked immunosorbent assay (ELISA) were present in 9/12 chickens in group B, whereas VLP‐vaccinated group A had no NP‐specific antibodies.

Two weeks after the booster vaccines, the pre‐challenge titres in group A (VLP‐vaccinated) had increased by 1–3 logs in 10/12 birds when tested against the 2016 antigen, with a GMT of 10.7 log_2_ (Table 1). The pre‐challenge HI titres in group B (commercial vaccine) similarly increased by 1 to 3 logs in all birds, with a GMT of 8.8 log_2_ as assessed against the homologous antigen. However, the GMT using the 2016 antigen was slightly lower at 8 log_2_, and this would be the more accurate predictor of protection, since the challenge strain was antigenically more similar to this test antigen. Apart from B6, all chickens in group B had increased levels of NP‐antibodies on ELISA, but ELISA values for group A remained negative, as expected, since the VLP does not contain NP.

Two weeks after challenge with the live replicating H6N2 virus, NP‐specific antibodies were present in 2/12 chickens in group A (A3 and A10), and the HI GMTs were slightly lower at 7.3 log_2_ and 9.9 log_2_ using the 2002 and 2016 test antigens, respectively (Table 1). NP antibody titres in group B remained similar to the pre‐challenge levels when GMTs are compared (S/N of 0.1 vs 0.2), but on HI, exposure to the 2016 challenge strain indicated increased antibodies detected by the 2016 test antigen (GMT of 8.8 log_2_) compared to the homologous test antigen (GMT of 8.1 log_2_). All non‐vaccinated chickens exposed to the challenge virus seroconverted with strong NP antibody responses (mean S/N of 0.27) and log_2_ HI titres of between 6 and 9 (GMT of 7.9 log_2_) against the 2016 test antigen, but only 6/12 chickens were positive on HI (GMT of 4.2 log_2_) against the 2002 antigen.

#### Challenge virus shedding

**Figure 3:**
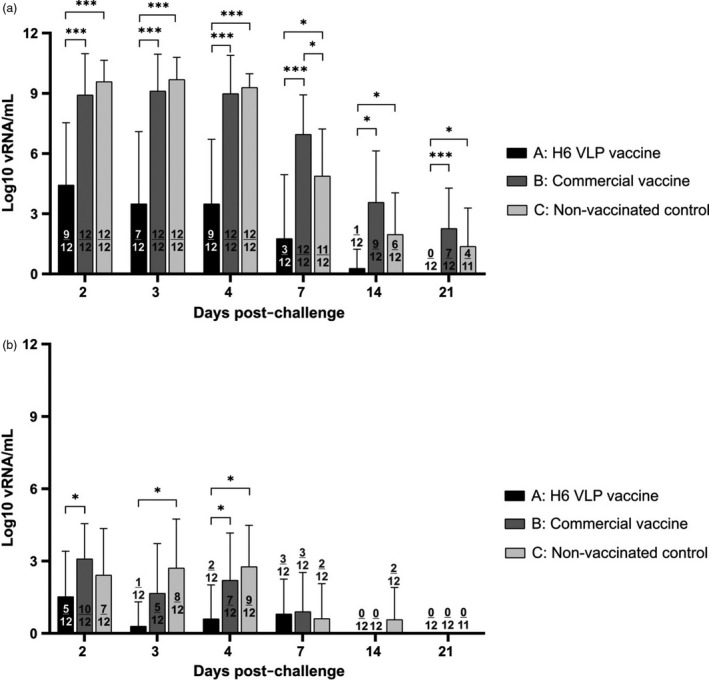
Virus shedding titres from the respiratory (a) and gastrointestinal (b) tracts following challenge with strain A/chicken/South Africa/H44954/2016 (H6N2), as assessed by qRT‐PCR. Statistical significance between mean titres at *P* < 0.05 (denoted by ‘*’) and *P* < 0.001 (denoted by ‘***’) was determined with Student's *t*‐test. The numbers of birds per group in which viral shedding was detected are indicated.

Oropharyngeal and cloacal swabs were collected from all chickens at days 2, 3, 4, 7, 14 and 21 post‐challenge, and the extracted nucleic acids were tested for the presence of the influenza A matrix gene by quantitative reverse transcription real‐time PCR (qRT‐PCR). In the H6 VLP‐vaccinated group A, 58% (7/12) of chickens were actively shedding virus from the oropharynx at day 3 post‐challenge (dpc) (Figure 3a, Table S2). Individuals A3 and A10 shed the highest amounts of 9.36 log_10_ and 9.28 log_10_ viral RNA (vRNA) copies/mL, respectively, whereas the group mean was considerably lower at 3.49 log_10_ vRNA copies/mL. At 4 dpc, the percentage of chickens shedding from the oropharynx had increased to 75% (9/12), with a slightly higher group mean of 3.78 log_10_ vRNA copies/mL (Figure 3a, Table S2). By 7 dpc, only 25% (3/12) of the chickens in this group were still shedding, with birds A2, A3 and A10's titres ranging from 6.23 to 7.54 log_10_ vRNA copies/mL. By 14 dpc, only a single bird (A2) was shedding, at the reduced level of 3.32 log_10_ vRNA copies/mL (Figure 3a, Table S2). The proportionately higher replication of challenge virus in birds A3 and A10 on days 2–7 post‐challenge correlates with the antibody responses detected at 14 dpc, as these were the only two birds in group A that had positive NP ELISA results, and their HI antibody titres were also among the highest at 12 log_2_. Three of the 12 chickens (A1, A6 and A7) had no detectable levels of virus in oropharyngeal swabs taken from 3 dpc onwards. Since their pre‐challenge antibody titres were among the lowest in the group at 9 or 10 log_2_, we presume that non‐evaluated cellular immune responses was responsible for the earlier cessation of viral shedding here.

In contrast, a larger proportion of chickens vaccinated with the commercial vaccine (group B) or non‐vaccinated (group C) shed significantly more virus from the oropharynx, for a longer period. In both groups B and C, 100% (12/12) of chickens shed high virus titres with group means >8.92 log_10_ until 4 dpc, with no statistical difference indicated (Figure 3a, Table S2). At 7 dpc, all of the chickens in group B (12/12) continued to shed virus with a mean group titre of 6.96 log_10_ vRNA copies/mL, whereas the group mean in non‐vaccinated birds (11/12) had reduced significantly to 4.87 log_10_ vRNA copies/mL (*P *<* *0.05). By 14 dpc, mean oropharyngeal shedding levels in groups B and C continued to decline in number to 3.57 and 1.96 log_10_ vRNA copies/mL, respectively, but 75% (9/12) of the chickens in vaccinated group B were still shedding compared with only 50% (6/12) in non‐vaccinated group C (Figure 3a, Table S2). At 21 dpc when the study ended, 58% (7/12) of chickens in vaccinated group B continued to shed virus from the respiratory tract (mean of 2.26 log_10_, vRNA copies/mL), in comparison with a lower 36% (4/11) of non‐vaccinated chickens (mean titre of 1.37 log_10_ vRNA copies/mL) (Figure 3a, Table S2). Interestingly, it appears that whereas the commercial vaccine initially reduced the mean post‐challenge oropharyngeal viral shed titres by 16.33%, it augmented viral shedding by 4.39% overall in comparison with the non‐vaccinated group (Table S2). Overall, the H6 VLP vaccine resulted in significantly less oropharyngeal shedding at each time point in comparison with the other treatment groups, with mean shedding reduced on average >100‐fold in comparison with the non‐vaccinated group, and >105‐fold compared to the commercial vaccine (calculated from egg infectious dose 50 (EID_50_) values in Table S2).

The total oropharyngeal shedding for each treatment group was between 5‐ and 6.5‐fold higher compared with cloaca viral titres in log_10_ terms (calculated from EID_50_ values in Tables S2 and S3), consistent with other published studies for LPAI in chickens (Arafat *et al*., 2018; Morales *et al*., 2009). However, the numerical proportion in EID_50_^′^s (Tables S2 and S3) is orders of magnitude greater; for example, a >5.7 million‐fold difference between oropharyngeal and cloacal shedding was determined at 2 dpc in group C. Cloacal shedding from H6 VLP‐vaccinated group A (Figure 3b, Table S3) was only detected in the first 7 days. Initially, 42% (5/12) of the H6 VLP‐vaccinated chickens were shedding with a group mean of 1.52 log_10_, vRNA copies/mL, but this dropped to 8% (1/12) at 3 dpc with a group mean of 0.29 log_10_ vRNA copies/mL, and then appeared to increase slightly to 2/12 birds at 4 dpc with a group mean of 0.6 log_10_, vRNA copies/mL. The shedding rose again at 7 dpc when 25% (3/12) of the chickens shed a mean of 0.8 log_10_ vRNA copies/mL, but shedding had ceased completely by 14 dpc (Figure 3b, Table S3). Overall, in the first 4 days post‐challenge, cloacal shedding in the VLP‐vaccinated group was reduced (descriptively at day 2; statistically at days 3 and 4; *P *<* *0.05) compared with the non‐vaccinated control group C. In group B, cloacal viral shedding was also detectable until 7 dpc, although the number of birds shedding was considerably higher (10/12, 5/12 and 7/12 for days 2, 3 and 4 post‐challenge, respectively) in comparison with group A, with an increase in viral titres ranging between 2.5‐ and 10‐ fold during this period (Table S3). At 7 dpc, 25% (3/12) of chickens vaccinated with the commercial vaccine shed detectable amount of virus with a group average of 0.9 log_10_ vRNA copies/mL, slightly higher than group A. Cloacal viral titres in group B were not significantly different from those of the non‐vaccinated control group C. In group C, viral excretion was detectable until 14 dpc in 17% (2/12) of the birds, with a mean titre of 0.57 log10 vRNA copies/mL (Figure 3b, Table S3). No virus was detected in the cloacal swabs of any group at 21dpc.

#### Clinical signs

No vaccine reactions were observed locally at the site of inoculation or systemically. No clinical signs or mortalities were observed in any birds after challenge, including the non‐vaccinated controls. At 16 dpc, bird C2 in the non‐vaccinated control group sustained an injury and was euthanized for humane reasons.

## Discussion

Abundant influenza H6‐subtype VLPs were transiently expressed in *N. benthamiana* leaves; the partially purified extract was emulsified with a commercial adjuvant and used to vaccinate SPF chickens. As per the standard practice in the field, chickens received a booster vaccine 4 weeks after the primary dose, and 2 weeks later, the birds were challenged with a live H6N2 field virus. Control groups included non‐vaccinated chickens and the only registered vaccine in South Africa, AVIVAC^®^ AI, a traditional egg‐grown whole inactivated H6N2 virus oil‐emulsion vaccine. A single dose of the plant‐produced H6 VLP vaccine elicited high H6‐specific antibodies in chickens, with no vaccine reactions. Remarkably, a single dose of the plant‐produced H6 VLP vaccine elicited an immune response (GMT of 9.3 log_2_) comparable to two doses of the commercial vaccine (GMT of 8.8 log_2_) as assessed against the respective closely related/homologous HI antigen, demonstrating the high potency of the H6 VLP vaccine.

As LPAI viruses do not induce clinical signs in disease‐free chickens under typical experimental conditions, the main determinant of vaccine efficacy is the ability to prevent or reduce viral shedding from the birds’ respiratory and gastrointestinal tracts. The H6 VLP vaccine reduced the shedding of a heterologous challenge virus by >100‐fold in the oropharynx and >6‐fold in the cloaca in comparison with non‐vaccinated chickens. Proportionately less H6 VLP‐vaccinated chickens shed virus from the oropharynx or cloaca compared to non‐vaccinated and control vaccine groups throughout the assessment phase, with viral shedding from the oropharynx ending at least a week sooner compared to the controls. Further reductions in shedding levels and duration might be expected had the H6 VLP vaccine and challenge strains been homologous. However, vaccines are never 100% identical to field strains and these results, therefore, provide a more realistic assessment of performance in the field.

The antibody response after vaccination with AVIVAC^®^ was strong and commensurate with the label claims of ≥6 log_2_, more so after the booster vaccination. However, the cost of the antigenic mismatch between the vaccine and challenge strains was evident in both the magnitude and duration of viral shedding, as well as the number of chickens that excreted virus. For the first 4 days following challenge, when the levels of virus excretion were at their highest, there was no reduction in shedding compared with the non‐vaccinated group. Alarmingly, a higher proportion of AVIVAC^®^‐vaccinated chickens shed greater quantities of virus from the respiratory tract than the non‐vaccinated control group from 7 dpc to 21 dpc when the study ended. We surmise that these viruses in the respiratory tract with enhanced replication capabilities are vaccine‐induced antigenic escape mutants and are planning follow‐up investigations.

In addition to the safety, potency and efficacy of the plant‐produced VLP vaccine, demonstrated here for the first time in chickens, plant‐produced influenza VLP vaccines for poultry have numerous other benefits over other traditional inactivated, subunit and live recombinant vaccines. Firstly, live recombinant, subunit and whole virus vaccines, whether derived from field isolates or by reverse genetics approaches, involve live viruses that must be handled under strict bio‐containment. Furthermore, SPF chicken eggs have supply and animal ethics considerations and vaccines involving bacterial, insect or mammalian cell cultures can be contaminated with endotoxins and pathogens (Moustafa *et al*., 2016). No live virus is employed at any stage in the production of plant‐produced VLPs, making plant‐produced VLP vaccines ethical, bio‐secure, sustainable and, due to the transient nature of expression, environmentally safe.

Differentiating infected from vaccinated animals (DIVA) compliance is of great importance in the field and is one of the characteristics of an ideal AI vaccine (Swayne and Kapczynski, 2017). Whole inactivated virus vaccines contain high levels of structural proteins (e.g. NP and matrix protein 1(M1)/M2) that elicit specific antibody responses in the host, and live replicating influenza virus field strains elicit immune responses against the full complement of viral proteins. We demonstrated strong NP antibody responses in chickens vaccinated with the commercial whole inactivated virus vaccine prior to challenge, whereas the NP ELISA results were consistently negative for the plant‐produced H6 VLP vaccine. The plant‐produced H6 VLP contains influenza M2 protein but not the NP, and no genetic material whatsoever. Vaccinated chickens can, therefore, be distinguished by applying a combination of appropriate serological tests. For example, the presence of HA‐ but absence of NP‐specific antibodies indicates a vaccine response, whereas the presence of both antibody types signifies exposure to a field virus. Alternatively, it is possible to engineer markers into the VLP for DIVA (Roy and Stuart, 2013).

Plant‐based expression is an ideal platform for producing veterinary vaccines due to low manufacturing costs, and the regulatory requirements for purity are less extensive in comparison with human vaccines (Meeusen *et al*., 2007). Although cost of a vaccine per bird is low, varying from $0.16 to $0.04 (WHO, 2012), the global poultry influenza vaccine market is massive. Of the >113 billion doses of the licensed commercial vaccines for HPAI H5 sold between 2002 and 2010, 95% were whole inactivated viruses, whereas the remaining 4.5% were live virus‐vectored vaccines or recombinant fowlpox virus vaccines (Swayne *et al*., 2014) and hundreds of millions of doses of LPAI H9N2 vaccines have been used across North Africa, the Middle East, and Central and South Asia in recent years (Jean Cilliers, Boehringer Ingelheim Inc., personal communication). A limitation of our study is that we did not perform a preliminary *in vivo* experiment to calculate the minimum effective dose but opted instead for a relatively high antigenic mass of 1:1024 (10 log_2_) or 768 HAU per dose. Just 40 g of infiltrated leaf material yielded enough H6 VLPs for 400 vaccine doses. Thus, conservatively estimated, more than 5000 chickens could be prime‐boost vaccinated per kilogram leaf material. In view of our results where a single immunization elicited excellent immune responses, 10 000 chickens could be immunized with one kg of leaf material, and a single vaccine with a long duration of immunity not only reduces other vaccination‐associated costs but minimizes handling and immunological stress on the birds. Kilany *et al*. (2016) determined that inactivated H9N2 vaccines containing at least 250 HAU/dose induced optimal protective titres and minimized virus shedding in SPF chickens. Our plant‐produced VLP vaccine contained 3‐fold more HA antigen; therefore, up to 30 000 chickens could potentially be vaccinated from just 1 kg of leaf material, although the efficacy of the lower antigenic mass dose would need to be verified *in vivo*.

Large‐scale vacuum infiltration and scalable purification (i.e. depth filtration for clarification followed by tangential flow filtration for ultrafiltration) is being tested with these H6 VLPs towards feasible commercial‐scale production. Commercial‐scale biopharming facilities in South Africa are in the initial planning phases, and the current onerous registration processes for conventional veterinary vaccines in South Africa and elsewhere need to be mitigated to allow the quick updating of AI vaccines. However, several Good Manufacturing Practice‐compliant biopharming facilities have been established globally and several plant‐based products (including VLP vaccines) for humans have already been approved by the Food and Drug Association or are in advanced clinical trials (Chen and Lai, 2013; Takeyama *et al*., 2015), paving the way for plant‐produced poultry vaccines.

With a growing demand for animal protein, coupled with rising concerns over animal welfare, microbial resistance to antibiotics and food safety, the focus in poultry health has switched from treatment to prevention. Controlling zoonotic infections at the source is particularly important from a One‐Health perspective. More efficacious antigen‐matched poultry vaccines are critical to curtailing the rapidly drifting field viruses, and therein lies the greatest advantage of HA‐based plant‐derived VLP vaccines: fully formulated influenza vaccines can be produced within 21 days of the availability of the HA sequence and scaled up to 10 million doses within a month (D'Aoust *et al*., 2010; Margolin *et al*., 2018). Looking further into the future, harnessing plant biotechnology to produce *in silico*‐predicted broadly protective influenza A vaccines (Ross *et al*., 2019) holds enormous promise for the future of AI control in poultry.

## Experimental procedures

### Synthetic clone design and plant expression vector construction

A synthetic codon‐optimized gene was designed based on the full HA protein sequence of sub‐lineage I strain A/chicken/South Africa/N2826/2016 (H6N2) (Figure S1). The gene was synthesized by Bio Basic Inc. (Toronto, ON, Canada) and contained the *Mus musculus* monoclonal antibody heavy chain variable region signal peptide sequence (O'Hara *et al*., 2012), with *Age*I and *Xho*I restriction enzyme recognition sites at the 5′‐ and 3′‐terminals, respectively, to allow cloning into the pEAQ‐HT plant expression vector (Sainsbury *et al*., 2012). Previous studies have shown that co‐expression with M1 is dispensable for VLP formation and even decreases VLP production in plants, whereas M2 improves influenza VLP production for some subtypes (Jutras *et al*., 2015). Therefore, a similar synthetic gene construct to the above but based on the M2 protein of strain A/New Caledonia/20/1999 (H1N1) (Genbank accession number HQ008884) that was already available at the CSIR was used.

The synthetic genes for H6 HA and M2 were cloned into pEAQ‐HT using the Fast‐Link DNA Ligase Kit (Epicentre, Madison, WI) according to manufacturer's instructions. Ligation mixtures were transformed into DH10B competent *Escherichia coli* cells via electroporation (Gene‐Pulser^™^ Bio‐Rad, Hercules, CA; 1.8 kV, 25 μF, 200 Ω). The bacterial cells were resuspended in 800 μL SOC broth (2% [w/v] tryptone, 0.5% [w/v] yeast extract, 10 mm NaCl, 2.5 mm KCl, 10 mm MgCl_2_, 20 mm MgSO_4_, and 20 mm glucose; pH 7.0), incubated for 1 h at 37 °C with agitation (200 r.p.m.) and plated onto Luria Bertani (LB) agar supplemented with 50 μg/mL kanamycin for overnight incubation at 37 °C. Selected clones were verified via colony PCR and DNA sequencing. PCRs were performed on the Mastercycler^®^ EP gradient S (Eppendorf, Hamburg, Germany) using the KAPA2G^™^ Robust PCR Kit (KAPA Biosystems, Cape Town, South Africa) and pEAQ‐HT specific primers (5′‐ACTTGTTACGATTCTGCTGACTTTCGGCGG‐3′; 5′‐CGACCTGCTAAACAGGAGCTCACAAAGA‐3′). The PCRs comprised of 4 μL of 5× KAPA 2G buffer, 0.4 μL dNTP mix, 0.4 μL (10 μm) of each primer, 0.1 μL of KAPA2G Robust DNA polymerase (5 U/μL), bacterial culture as template and sterile nuclease‐free water to a final volume of 20 μL. The cycling conditions entailed 1 cycle at 95 °C for 2 min, 35 cycles of 95 °C for 15 s, 60 °C for 30 s and 72 °C for 45 s, with a final extension step of 72 °C for 5 min. Following electrophoretic separation, PCR positive clones were inoculated into LB media for plasmid DNA isolation (Zyppy^™^ Plasmid Miniprep Kit; Zymo Research, Irvine, CA), and DNA was submitted to Inqaba Biotec (Pty) Ltd. (Pretoria, South Africa) for Sanger sequencing.

### Agroinfiltration of *N. benthamiana* with transformed *Agrobacterium tumefaciens*

*Agrobacterium* strain AGL‐1 was obtained from the American type culture collection (ATCC^®^ BAA‐101TM, *Rhizobium radiobacter*). Sequence‐verified pEAQ‐HT+H6 and pEAQ‐HT+M2 plasmids were transformed into *Agrobacterium tumefaciens* cells using electroporation (1.44 kV, 25 μF, 200 Ω). The bacterial cells were resuspended in 800 μL LB and incubated for 3 h at 28 °C with agitation (200 r.p.m.), plated onto LB agar supplemented with selective antibiotics (30 μg/mL rifampicin, 50 μg/mL kanamycin and 50 μg/mL carbenicillin) and incubated over 48 h at 28 °C. Antibiotic‐resistant clones were selected for verification via colony PCR as before.

The leaves of 5‐ to 8‐week‐old *N. benthamiana* plants modified to allow mammalian‐like glycosylation (Strasser *et al*., 2008) were infiltrated with validated transformed *A. tumefaciens* clones (Shamloul *et al*., 2014). *A. tumefaciens* cultures containing pEAQ‐HT+H6 and pEAQ‐HT+M2, respectively, were subcultured and grown overnight at 28 °C in LB containing 30 μg/mL rifampicin and 50 μg/mL kanamycin, pelleted by centrifugation at 8000 *g* for 8 min and resuspended in infiltration buffer (10 mm 2‐N‐morpholino‐ethanesulfonic acid (MES), 20 mm MgSO_4_, pH 5.6) containing 200 μm acetosyringone. The respective infiltration mixes were diluted to obtain a final optical density at 600 nm of 1.5 and mixed to contain equal parts pEAQ‐HT+H6 and pEAQ‐HT+M2 plasmids. Following incubation at room temperature for 1 h, the *A. tumefaciens* suspension was introduced into the leaves by hand, using a syringe without a needle.

### VLP extraction, purification and confirmation of expression

Six days after infiltration, 40 g of infiltrated leaves was harvested and homogenized in two volumes of buffer [50 mm tris(hydroxymethyl)aminomethane (Tris), 150 mm NaCl, and 0.04% sodium metabisulfite, pH 8.0) (Landry *et al*., 2010)] supplemented with proteinase inhibitor cocktail (P2714; Sigma‐Aldrich) using a Matstone DO9001 Juicer. The homogenate was clarified through cheese cloth and purified using differential centrifugation. The clarified extract was loaded onto an Iodixanol (OptiPrep^™^; Sigma‐Aldrich) density gradient ranging from 20% to 60%, followed by ultracentrifugation (32 000 *g*, 2 h, 10 °C; Beckman Coulter Ultra‐centrifuge Optima L90K). Fractions were collected from the bottom of the Thinwall Ultra‐Clear^™^ tube (Beckman Coulter, Brea, CA), and the three fractions containing the most abundant H6 protein, as determined using SDS‐PAGE and immunoblotting, were pooled. Following dialysis in phosphate‐buffered saline (PBS, pH 7.4) using SnakeSkin Dialysis Tubing (10K MWCO, 35 mm dry I.D.; Thermo Scientific, Waltham, MA), trehalose dihydrate (15% w/v) (Sigma‐Aldrich) was added.

Partially purified plant extract was separated on an Invitrogen Bolt^™^ 4%–12% Bis‐Tris Plus gel (Thermo Scientific) under reducing conditions and stained with Coomassie G‐250. In previous experiments, partially purified VLP proteins were quantified using the Micro BCA^™^ Protein Assay Kit (Thermo Scientific) according to the manufacturer's instructions, using Bovine Gamma Globulin (Bio‐Rad) as a protein standard. The SDS‐PAGE protein band corresponding to the expected HA fragment size (approximately 62 kDa) was excised and in‐gel trypsin digested (Shevchenko *et al*., 2007) for analysis by LC‐MS/MS‐based peptide sequencing at CSIR Biosciences, Pretoria, as described by Chhiba‐Govindjee *et al*. (2018).

Proteins were separated on a 10% TGX Stain‐Free^™^ FastCast^™^ acrylamide gel (Bio‐Rad) under reducing conditions and transferred to an Immobilon PVDF membrane using the transblot turbo blotter (Bio‐Rad), according to manufacturer's recommendation. Blocking was performed in 1× PBS containing 0.1% Tween 20 (Merck, Darmstadt, Germany) and 3% Bovine Serum Albumin Fraction V (Sigma‐Aldrich) for 2 h. H6N2 antiserum (1:600 dilution; Deltamune (Pty) Ltd.) was added as the primary antibody and incubated on a rotary shaker for 2 h, followed by washing with 1× PBS. Goat anti‐chicken IgY horseradish peroxidase conjugated antibody (1:1500) (Novex Life Technologies, Thermo Fisher Scientific) was added and incubated on a rotary shaker for 2 h. After final washing with 1× PBS, proteins were visualized using chemiluminescence detection (Clarity^™^ Western ECL Blotting Substrate; Bio‐Rad) on the ChemiDoc^™^ MP Imaging System (Bio‐Rad), according to manufacturer's instructions.

Partially purified H6‐VLP preparations were examined by microscopy at the Electron Microscopy Unit, University of Pretoria (UP). Carbon‐coated copper grids (mesh size 200) were floated on 15 μL density gradient fractions for 5 min and washed by floating on 5 μL sterile water, five times. Particles were negatively stained for 30 s with 2% uranyl acetate and imaged using a Philips CM10, 80 kV transmission electron microscope.

### Efficacy study in chickens

#### Experimental animals

The vaccine‐challenge study in chickens (*Gallus gallus*) was carried out in the Veterinary Faculty's Poultry Biosafety Level 3 facility. All procedures were pre‐approved by the Animal Ethics and Research Ethics Committees of the University of Pretoria and the CSIR Research Ethics Committee. Six‐week‐old SPF White Leghorn type chickens (*n* = 36) purchased from Avi‐Farms (Pty) Ltd., Pretoria, South Africa were numbered individually and randomly assigned in isolators into three treatment groups. Layer grower feed (Nova Feeds, Pretoria, South Africa) and water was provided *ad libitum* for the duration of the trial.

#### Vaccines

Partially purified H6 VLPs were tested by hemagglutination and HI assays (see Serological Testing) and stored at 4 °C until use. The vaccine dose of 35.7 μL of plant leaf extract was calculated to correspond to an HI titre of 1:1024 (10 log_2_) or 768 HAU. On the day of vaccination, the partially purified plant‐produced H6 VLPs diluted in PBS were mixed in a 1:1 ratio with Montanide^™^ ISA 71 VG adjuvant (Seppic, Paris, France).

The commercially inactivated H6N2 oil‐emulsion vaccine (AVIVAC^®^ AI) with batch No. 60076 and expiration date of 05/2019 was purchased under a Department of Agriculture, Forestry and Fisheries (DAFF) Section 20 permit from the manufacturer. The vaccine seed strain is A/Chicken/South Africa/W‐04/2002(H6N2), a sub‐lineage I virus (Rauff *et al*., 2016). According to the label, the EID_50_ of the commercial vaccine is ≥10^8^ per recommended dose (0.5 mL) and results in a high immune response (HI titre ≥6 log_2_).

#### Challenge virus

The field strain used in the design of the H6 VLP vaccine, viz. A/chicken/South Africa/N2826/2016 (H6N2), could not be used for challenge because the isolate, cultured at UP from a flock infected with multiple pathogens, was contaminated with a virulent Newcastle disease virus. Instead, strain A/chicken/South Africa/H44954/2016 (H6N2), also a sub‐lineage I virus, was obtained from (RCL Foods (Pty) Ltd., Centurion, South Africa). This virus was isolated from tracheal samples of 56‐week‐old commercial layer hens in Pietermaritzburg, KwaZulu‐Natal Province in November 2016. The flock had been vaccinated with the AVIVAC^®^ vaccine but still showed a 10% drop in egg production. Mild tracheitis and secondary *E. coli* peritonitis and airsacculitis were seen on post‐mortem. A/chicken/South Africa/H44954/2016 (H6N2) shares 95.77% amino acid sequence identity with strain A/chicken/South Africa/N2826/2016 in the HA protein (Table S1). The challenge virus was propagated further at UP in SPF embryonated chicken eggs, and the EID_50_ was determined according to the method of Reed and Muench (1938). Stock with a titre of 10^6.8^ EID_50_ was aliquoted and frozen at −80 °C until use. On the day of challenge, stock was thawed and diluted in OculoNasal diluent (Intervet) to 10^6^ EID_50_/0.06 mL, corresponding to one drop in each eye. The prepared challenge material was kept on ice until administered.

#### Experimental design

At day 0 of the study, 1 mL blood each was sampled from the wing vein of 10 randomly selected chickens to confirm that the SPF chickens had no prior exposure to influenza A virus. Group A (*n* = 12) was vaccinated intra‐muscularly in the breast with 0.3 mL of the H6 VLP vaccine, while group B (*n* = 12) was vaccinated intra‐muscularly in the breast with 0.5 mL of the commercial H6N2 vaccine. Twenty‐eight days after the primary vaccination, all chickens in groups A and B were bled as above and subsequently received a booster of the respective vaccine. Fourteen days after administration of the booster vaccine, all vaccinated chickens were bled and all birds in groups A, B and C were subsequently challenged with 10^6^ EID_50_ of the challenge virus via the oculonasal route. Chickens were observed daily throughout the trial for adverse vaccine effects and after challenge for clinical signs of disease such as conjunctivitis, ocular or nasal discharge, respiratory distress such as difficulty breathing, coughing or snicking, loss of appetite, huddling, ruffled feathers or general depression. At days 2, 3, 4, 7, 14 and 21 after viral challenge, sterile plastic applicator rayon‐tipped swabs (Copan) were used to swab the cloaca and the choanal clefts of each chicken. Swabs were placed individually into 1 mL of viral transport media (VTM; brain–heart broth, 0.1 mg/mL doxycycline, 0.1 mg/mL enrofloxacin, 1 mg/mL penicillin–streptomycin and 10% glycerol) and kept at 4 °C until processing. Blood was drawn again at 14 dpc, and chickens were humanely euthanized at 21 dpc.

#### Serological testing

Blood was incubated at room temperature for at least an hour, centrifuged at 5000 *g* for 10 min at 4 °C and the sera transferred to sterile tubes. NP‐specific antibodies that are antigenically conserved served among influenza A viruses (OIE, 2018) were detected with IDEXX Influenza A virus Antibody test kits according to manufacturer's instructions, using an iMark^™^ Microplate Reader (Bio‐Rad). The sample to negative control ratio (S/N) was calculated from the optical density at A^655^ for each sample. A S/N < 0.5 is considered as positive. Sera were also submitted to the veterinary diagnostic laboratory of the University's Department of Veterinary Tropical Diseases where hemagglutination and HI assays were performed according to the OIE‐recommended procedures (OIE, 2018). Two antigens were used for HI testing, A/chicken/South Africa/W‐04/2002 (H6N2), homologous with AVIVAC^®^ AI (Rauff *et al*., 2016), and challenge virus A/chicken/South Africa/H44954/2016 (H6N2). The H6 VLP and the challenge strain were only 95.77% identical in the HA protein (Table S1). The H6 VLP's homologous live virus, A/chicken/South Africa/N2826/2016 (H6N2), could not be used as HI test antigen because it was contaminated with a virulent Newcastle disease virus. The latter also agglutinates erythrocytes, and it would therefore obfuscate the accurate estimation of HA units. HI titres were considered to be positive if complete inhibition of hemagglutination was observed at a sample dilution of 1:16 (2^4^ or 4 log_2_) or more.

#### Viral detection by real‐time quantitative reverse transcription PCR

Total RNA was extracted from swab fluids using TRIzol^™^ Reagent (Thermo Fisher Scientific) according to the recommended procedure. RNAs were tested for the presence of the influenza A virus group in a qRT‐PCR protocol that targets the conserved matrix protein gene, using the primers and probes described by Spackman *et al*. (2003). The qRT‐PCRs were carried out on the StepOnePlus^™^ platform (Life Technologies, Thermo Fisher Scientific) using VetMax^™^‐Plus One‐Step RT‐PCR Kits (Life Technologies). Each qRT‐PCR consisted of the following: 3 μL RNA, 6 μL 2 × RT‐PCR buffer, 0.5 μL 25 × RT‐PCR enzyme mix, 0.5 μL of each primer (10 μm), 0.15 μL probe (5 μm), and PCR grade water to a final volume of 12 μL. Cycling conditions entailed 1 cycle of 48 °C for 10 min, 1 cycle of 95 °C for 10 min and 40 cycles of 95 °C for 15 s followed by 53 °C for 45 s. Tenfold serial dilutions of RNA extracted from the viral challenge were used to generate a standard curve for calculating the relative EID_50_ quantity in each sample. Samples with a cycle threshold (Ct) value of less than 40 were considered positive. To calculate the viral RNA copy numbers, a serial dilution of the EID_50_ ‐titrated control RNA was used to determine the lowest limit of detection for the M‐gene qRT‐PCR assay which was 10^−8^, and the EID_50_ value obtained was divided by the empirically determined limit of detection of 1000 viral RNA copies (Spackman *et al*., 2003) to determine a factor of 51 398.03 by which every test RNA EID_50_ value was multiplied.

### Statistical analysis and graphs

Viral RNA and antibody titres among groups were analysed using one‐way analysis of variance (ANOVA). Pairwise mean comparisons between groups were analysed using Student's *t*‐test. A *P*‐value of ≤0.05 was considered as significant. All graphs were generated using (GraphPad Prism 8.0.2, San Diego, CA).

## Conflict of interest

The authors declare no conflicts of interest.

## Author contributions

CA and MOK conceived the project; CA, MOK and TS designed the plant experiments; TS performed the plant experiments. CA and DBRW designed the animal experiments; TS, DBRW, MA and CA performed the animal experiments. CA, TS and MOK wrote the manuscript with assistance from the other authors.

## Supporting information


**Figure S1** Multiple sequence alignment of the hemagglutinin (HA) proteins of the strains used in this study.

**Figure S2** LC‐MS/MS‐based peptide sequence analysis for SDS‐PAGE bands of approximately 62 kDa (A) and 14 kDa (B), respectively.

**Table S1** Pairwise amino acid distances of the hemagglutinin proteins of H6N2 strains used in the study.

**Table S2** qRT‐PCR results for oropharyngeal swabs as log10 vRNA viral titres/mL, with EID_50_/mL titres in parenthesis.

**Table S3** qRT‐PCR results for cloacal swabs as log_10_ vRNA viral titres/mL, with EID_50_/mL titres in parenthesis.
