# Supplementary material for: Efficacy of a plant‐produced virus‐like particle vaccine in chickens challenged with Influenza A H6N2 virus
Source: Plant Biotechnol J. 2019 Aug 22;18(2):502–12. doi: 10.1111/pbi.13219 (PMC6953208; doi:10.1111/pbi.13219)
Supplement: Supplementary file 1 — Figure S1 Multiple sequence alignment of the hemagglutinin (HA) proteins of the strains used in this study. [file PBI-18-502-s004.pdf]

```
                                                 10        20        30        40        50        60        70        80        90       100
                                        ....|....|....|....|....|....|....|....|....|....|....|....|....|....|....|....|....|....|....|....|
Consensus                               MIAII--A-L-STGKSDKICIGYHANNSTTQVDTILEKN-TVTHSIELLE-QKEERFC--LNKAPLDLRECTIEGW-LGNP-CD-LL-DQ-WSYIVERP-
A/chicken/South Africa/W04/2002(H6N2)   .....VM.I.A............................V..........N.......KI................I....Q..L..G..S........T
A/chicken/South Africa/N2826/2016(H6N2) .....AI.L.V............................I..........T.......RV................M....R..I..E..R........S
A/chicken/South Africa/H44954/2016(H6N2) .....AI.L.V............................I..........T.......RI................M....Q..I..E..R........S

                                                110       120       130       140       150       160       170       180       190       200
                                        ....|....|....|....|....|....|....|....|....|....|....|....|....|....|....|....|....|....|....|....|
Consensus                               A-NGICYPGPLNE-EEL--LIGSGERVERFEMFPKSTW-GVDT--GI--ACSS-TGGSSFYRNLLWIIKNKSA-YPVIKGTYNNTG-QPI-YFWGVHHPP
A/chicken/South Africa/W04/2002(H6N2)   .Q...........V...KA...................T....DS..TK....-...................A............N...L.........
A/chicken/South Africa/N2826/2016(H6N2) .S...........I...RS...................N....EN..TR....S...................S............N...I.........
A/chicken/South Africa/H44954/2016(H6N2) .Q...........V...RS...................T....DN..TI....S...................S............K...I.........

                                                210       220       230       240       250       260       270       280       290       300
                                        ....|....|....|....|....|....|....|....|....|....|....|....|....|....|....|....|....|....|....|....|
Consensus                               D---QNNLYGSGDRY-RMGTESM-FAK-PEIAA-P-VNGQRGRIDYYWS-LNPGETLN-ESNGN-IAPRYAYRFFS-N-KG--FKSNLPIENCDA-CQTT
A/chicken/South Africa/W04/2002(H6N2)   .TNG...........V.......N...S.....R.A.............V........V.....L...........T.N..AV............L....
A/chicken/South Africa/N2826/2016(H6N2) .ADR...........I.......H...G.....R.S.............V........I.....F...........T.K..VI............Q....
A/chicken/South Africa/H44954/2016(H6N2) .ADR...........I.......H...S.....G.S.............I........V.....F...........S.K..II............Q....

                                                310       320       330       340       350       360       370       380       390       400
                                        ....|....|....|....|....|....|....|....|....|....|....|....|....|....|....|....|....|....|....|....|
Consensus                               -GVLRTNKTFQNVSP-W-GECPKYVKSKSLRLATGLRN-PQ--TRG-FGAIAGFIEGGWTGMIDGWYGYHHENSQGSGYAADR-STQKAIDGITNKVN-I
A/chicken/South Africa/W04/2002(H6N2)   A..............L.I....................V..IE...L....................................E..............A.
A/chicken/South Africa/N2826/2016(H6N2) L..............Q.T....................V..VE...I....................................D..............T.
A/chicken/South Africa/H44954/2016(H6N2) S..............Q.I....................I..VG...I....................................D..............S.

                                                410       420       430       440       450       460       470       480       490       500
                                        ....|....|....|....|....|....|....|....|....|....|....|....|....|....|....|....|....|....|....|....|
Consensus                               IDKMNTQFEAV-HEFSNLERRIDN-NKRMEDG-LDVWTYNAELLVLLENERTLDLHDANVKNLYE-VKSQLRDNANDLGNGCFEFWHKCDNDCMESVKNG
A/chicken/South Africa/W04/2002(H6N2)   ...........D............L.......F................................K..................................
A/chicken/South Africa/N2826/2016(H6N2) ...........G............L.......L................................R..................................
A/chicken/South Africa/H44954/2016(H6N2) ...........G............M.......L................................K..................................

                                                510       520       530       540       550       560
                                        ....|....|....|....|....|....|....|....|....|....|....|....|....|..
Consensus                               TYDYPKYQDESKLNRQ-IESV-LDNLGVYQILAIYSTVSSSLVLVGLI-AMGLWMCSNGSMQCR-CI
A/chicken/South Africa/W04/2002(H6N2)   ................E....K..........................I...............I..
A/chicken/South Africa/N2826/2016(H6N2) ................K....K..........................I...............V..
A/chicken/South Africa/H44954/2016(H6N2) ................K....N..........................M...............I..
```

Figure S1. Multiple sequence alignment of the hemagglutinin (HA) proteins of the strains used in this study. W-04 is the AVIVAC H6N2 vaccine seed strain, N2826 was used in the design of the plant-produced VLP vaccine, and H44954 was the challenge virus. Sequences are aligned to a consensus with identical residues plotted with a dot.
