# Supplementary material for: Efficacy of a plant‐produced virus‐like particle vaccine in chickens challenged with Influenza A H6N2 virus
Source: Plant Biotechnol J. 2019 Aug 22;18(2):502–12. doi: 10.1111/pbi.13219 (PMC6953208; doi:10.1111/pbi.13219)
Supplement: Supplementary file 2 — Figure S2 LC‐MS/MS‐based peptide sequence analysis for SDS‐PAGE bands of approximately 62 kDa (A) and 14 kDa (B), respectively. [file PBI-18-502-s003.pdf]

A

DKICIGYHANNSTTQVDTILEKNITVTHSIELLETQKEERFCRVLNKAPLDLR**ECTIEGWMLGNPRCDILLEDQR**
WSYIVERPSASNGICYPGPLNEIEELR**SLIGSGERVERFEMFPKSTWNGVDTENGITR**ACSSSTGGSSFYR**NLLW**
IIKNK**SASYPVIK**GTYNNTGNQPIIYFWGVHHPPDADR**QNNLYGSGDR**YIR**MGTESMHFAKGPEIAARPSVNGQR**
GRIDYYWSVLNPGETLNIESNGNFIAPRYAYR**FFSTNKK**GVIFKSNLPIENCDAQCQTTLGVLRTNK**TFQNVSPQ**
WTGECPKYVKSKSLRLATGLR**NVPQVETR**GIFGAIAGFIEGGWTGMIDGWYGYHHENSQGSGYAADR**DSTQKAID**
GITNKVNTIIDKMNTQFEAVGHEFSNLERRIDNLNKRMEDGLLDVWTYNAELLVLLENER**TLDLHDANVKNLYER**
VKSQLR**DNANDLGNGCFEFWHK**CDNDCMESVK**NGTYDYPK**YQDESKLNR**QKIESVK**LDNLGVYQILAIYSTVSSS
LVLVGLIIAMGLWMCSNGSMQCRVCI

B

MSLLTEVETPIRNEWGCRCNDSSDPLVVAASIIGIVHLILWIIDRLFSKSIYRIFKHGLK**RGPSTEGVPESMREE**
YREEQQNAVDADDGHFVSIELE

Figure S2. LC-MS/MS based peptide sequence analysis for SDS-PAGE bands of approximately 62 kDa (A) and 14 kDa (B), respectively. For the 62 kDa band, peptide sequences are mapped against the hemagglutinin protein sequence of A/chicken/South Africa/N2826/2016 (H6N2) for reference. For the 14 kDa band, peptide sequences are mapped against the M2 ion channel protein sequence of A/New Caledonia/20/1999 (H1N1) for reference. Peptides identified with >95% confidence are highlighted in green and <50% confidence in red. No peptides were identified for the non-highlighted grey regions of the protein sequences.
