# Supplementary material for: Efficacy of a plant‐produced virus‐like particle vaccine in chickens challenged with Influenza A H6N2 virus
Source: Plant Biotechnol J. 2019 Aug 22;18(2):502–12. doi: 10.1111/pbi.13219 (PMC6953208; doi:10.1111/pbi.13219)
Supplement: Supplementary file 3 — Table S1 Pairwise amino acid distances of the hemagglutinin proteins of H6N2 strains used in the study. [file PBI-18-502-s005.pdf]

Table S1. Pairwise amino acid distances of the hemagglutinin proteins of H6N2 strains used in the study

Strain	W04	N2826	H44954
A/chicken/South Africa/W-04/2002<br>*AVIVAC vaccine seed strain*		91.18%	91.53%
A/chicken/South Africa/N2826/2016<br>*Plant-produced VLP H6*			95.77%
A/chicken/South Africa/H44954/2016<br>*Challenge strain*			
