# Supplementary material for: Efficacy of a plant‐produced virus‐like particle vaccine in chickens challenged with Influenza A H6N2 virus
Source: Plant Biotechnol J. 2019 Aug 22;18(2):502–12. doi: 10.1111/pbi.13219 (PMC6953208; doi:10.1111/pbi.13219)
Supplement: Supplementary file 4 — Table S2 qRT‐PCR results for oropharyngeal swabs as log10 vRNA viral titres/mL, with EID50/mL titres in parenthesis. [file PBI-18-502-s001.pdf]

Table S2: qrRT-PCR results for oropharynageal swabs as log_{10} vRNA viral titres/ml, with EID_{50}/ml titres in parenthesis

Treatment group	Chicken No.	Sampling day post challenge 2	3	4	7	14	21
A: H6 VLP vaccine	A1	3.40 [0.05]	U	U	U	U	U
	A2	6.80 [122.80]	6.65 [87.89]	6.63 [83.08]	6.23 [33.05]	3.32 [0.04]	U
	A3	8.56 [7,087.50]	9.36 [44,166.00]	8.49 [5,972.72]	7.30 [390.38]	U	U
	A4	3.74 [0.11]	3.65 [0.09]	4.07 [0.23]	U	U	U
	A5	U	5.48 [5.91]	3.24 [0.03]	U	U	U
	A6	4.51 [0.63]	U	U	U	U	U
	A7	U	U	U	U	U	U
	A8	U	3.68 [0.09]	3.65 [0.09]	U	U	U
	A9	5.80 [12.29]	3.75 [0.11]	3.44 [0.05]	U	U	U
	A10	8.48 [5,882.91]	9.28 [36,683.59]	8.95 [17,352.63]	7.54 [669.72]	U	U
	A11	5.93 [16.57]	U	3.32 [0.04]	U	U	U
	A12	5.94 [17.02]	U	3.52 [0.06]	U	U	U
	Mean	4.43 ± 3.10 [1,094.99 ± 2531.06]	3.49 ± 3.60 [6,745.31 ± 15,812.36]	3.78 ± 3.03 [1,950.75 ± 5,144.54]	1.76 ± 3.19 [91.10 ± 231.72]	0.28 ± 0.96 [0.00 ± 0.01]	U
B: Commercial H6N2 vaccine	B1	8.95 [17,147.34]	9.20 [31,123.68]	9.47 [56,762.66]	7.82 [1,271.09]	4.07 [0.23]	3.30 [0.04]
	B2	9.21 [31,693.99]	9.03 [208,57.23]	8.98 [18,794.16]	5.67 [9.15]	4.66 [0.89]	U
	B3	4.44 [0.54]	5.47 [5.72]	5.68 [9.35]	3.96 [0.18]	U	U
	B4	8.69 [9,496.08]	10.06 [222,982.17]	10.38 [465,251.66]	7.35 [436.86]	5.20 [3.08]	3.82 [0.13]
	B5	10.18 [294,594.91]	10.72 [1,011,907.56]	10.02 [204,423.14]	9.29 [38,369.80]	3.51 [0.06]	U
	B6	5.38 [4.67]	5.57 [7.21]	4.80 [1.22]	3.63 [0.08]	U	U
	B7	8.40	8.33	7.97	5.09	3.78	3.77

		[4,941.30]	[4,172.49]	[1,808.42]	[2.38]	[0.12]	[0.11]
	B8	9.68<br>[94,055.52]	9.29<br>[38,088.14]	10.53<br>[653,765.94]	9.25<br>[34375.70]	8.87<br>[14,506.68]	U
	B9	11.26<br>[3,554,684.50]	10.47<br>[573,779.94]	9.41<br>[49,937.49]	7.49<br>[607.32]	3.82<br>[0.13]	4.43<br>[0.53]
	B10	10.83<br>[1,319,418.75]	10.73<br>[1,053,922.00]	10.51<br>[631,874.00]	8.46<br>[5,616.02]	4.71<br>[1.00]	3.99<br>[0.19]
	B11	9.71<br>[100,821.03]	9.86<br>[141,862.56]	9.67<br>[90,377.52]	6.73<br>[105.34]	U	3.55<br>[0.07]
	B12	10.25<br>[342,713.22]	10.63<br>[838,345.88]	10.39<br>[47,4669.69]	8.72<br>[10,240.57]	4.22<br>[0.32]	4.26<br>[0.36]
	Mean	8.92 ± 2.06<br>[480,797.65 ±<br>1,036,923.51]	9,11 ± 1.84<br>[328,087.88 ±<br>420,797.26]	8.98 ± 1.91<br>[220,639.60 ±<br>259,421.07]	6.96 ± 1.96<br>[7,586.21 ±<br>13,823.30]	3.57 ± 2.56<br>[1,209.38 ±<br>4,187.56]	2.26 ± 2.02<br>[1.45 ± 0.12]
C:<br>Non-<br>vaccinated<br>control	C1	9.83<br>[132,184.05]	9.92<br>[162,635.92]	9.20<br>[30,582.46]	5.78<br>[11.67]	3.76<br>[0.11]	U
	C2	9.07<br>[22,873.56]	10.12<br>[255,302.47]	8.99<br>[19,051.86]	U	U	†
	C3	11.03<br>[2,094,932.25]	10.73<br>[1,052,160.00]	10.80<br>[1,229,791.88]	9.61<br>[79,515.58]	4.75<br>[1.08]	U
	C4	10.83<br>[1,308,116.13]	10.27<br>[366,290.41]	9.69<br>[95,887.45]	7.33<br>[416.87]	3.65<br>[0.09]	3.68<br>[0.09]
	C5	9.06<br>[22,264.40]	9.57<br>[72,389.12]	9.69<br>[96,366.26]	5.31<br>[4.00]	4.05<br>[0.22]	4.26<br>[0.36]
	C6	11.17<br>[2,880,247.00]	10.04<br>[212,548.03]	9.58<br>[74,106.70]	4.52<br>[0.64]	U	U
	C7	9.18<br>[29,331.09]	9.57<br>[72,102.30]	9.30<br>[39,215.80]	3.63<br>[0.08]	U	U
	C8	8.40<br>[4,852.83]	9.39<br>[47,933.50]	8.66<br>[8,931.28]	3.70<br>[0.10]	U	U
	C9	9.09<br>[23,994.57]	6.42<br>[50.74]	8.32<br>[4,092.66]	4.01<br>[0.20]	U	U
	C10	10.24<br>[335,462.56]	9.61<br>[79,864.50]	8.50<br>[6,085.93]	4.00<br>[0.19]	3.33<br>[0.04]	3.65<br>[0.09]
	C11	9.31<br>[40,149.06]	10.03<br>[208,241.86]	8.91<br>[15,671.43]	4.19<br>[0.30]	4.02<br>[0.20]	3.51<br>[0.06]
	C12	7.71<br>[1,002.83]	10.53<br>[654,263.75]	9.84<br>[133,386.89]	6.42<br>[51.62]	U	U
	Mean	9.58 ± 1.07<br>[574,617.53 ±<br>980,148.75]	9.68 ± 1.11<br>[265,315.22 ±<br>305,085.74]	9.29 ± 0.69<br>[146,097.55 ±<br>343,938.97]	4.87 ± 2.35<br>[6,666.77 ±<br>22,941.73]	1.96 ± 2.08<br>[0.15 ±<br>0.31]	1.37 ± 1.91<br>[1.12 ±<br>0.05]

U-undetected/ below the limit of detection of 1000 viral copies ; † chicken C2 was euthanized 16 days post challenge for humane reasons unrelated to viral challenge
