# Supplementary material for: Efficacy of a plant‐produced virus‐like particle vaccine in chickens challenged with Influenza A H6N2 virus
Source: Plant Biotechnol J. 2019 Aug 22;18(2):502–12. doi: 10.1111/pbi.13219 (PMC6953208; doi:10.1111/pbi.13219)
Supplement: Supplementary file 5 — Table S3 qRT‐PCR results for cloacal swabs as log10 vRNA viral titres/mL, with EID50/mL titres in parenthesis. [file PBI-18-502-s002.pdf]

Table S3: qrRT-PCR results for cloacal swabs as log_{10} vRNA viral titres/ml, with EID_{50}/ml titres in parenthesis

Treatment group	Chicken No.	Sampling day post challenge					
		2	3	4	7	14	21
A: H6 VLP vaccine	A1	3.85 [0.14]	3.51 [0.06]	U	U	U	U
	A2	U	U	U	U	U	U
	A3	U	U	3.92 [0.16]	3.46 [0.06]	U	U
	A4	U	U	U	U	U	U
	A5	3.37 [0.05]	U	3.29 [0.04]	U	U	U
	A6	3.68 [0.09]	U	U	U	U	U
	A7	3.48 [0.06]	U	U	U	U	U
	A8	3.90 [0.15]	U	U	U	U	U
	A9	U	U	U	U	U	U
	A10	U	U	U	3.07 [0.02]	U	U
	A11	U	U	U	3.09 [0.02]	U	U
	A12	U	U	U	U	U	U
	Mean	1.52±1.89 [0.04 ± 0.06]	0.29±1.01 [0.01 ± 0.02]	0.60±1.41 [0.02 ± 0.05]	0.80±1.45 [0.01 ± 0.02]	U	U
B: Commercial H6N2 vaccine	B1	3.69 [0.10]	3,88 [0.15]	U	U	U	U
	B2	3.68 [0.09]	U	3.41 [0.05]	U	U	U
	B3	3.62 [0.08]	U	U	U	U	U
	B4	3.83 [0.13]	3,73 [0.10]	3.46 [0.06]	U	U	U
	B5	U	3,77 [0.11]	4.19 [0.30]	U	U	U
	B6	3.48 [0.06]	U	3.46 [0.06]	U	U	U
	B7	4.34 [0.42]	U	U	U	U	U

	B8	3.63<br>[0.08]	4,50<br>[0.61]	4.13<br>[0.26]	U	U	U
	B9	3.50<br>[0.06]	U	U	U	U	U
	B10	3.48<br>[0.06]	U	4.03<br>[0.21]	4.05<br>[0.22]	U	U
	B11	U	U	U	3.26<br>[0.04]	U	U
	B12	3.82<br>[0.13]	4,08<br>[0.23]	3.77<br>[0.12]	3.45<br>[0.06]	U	U
	Mean	3.09±1.46<br>[0.10 ± 0.11]	1.66±2.06<br>[0.10 ± 0.18]	2.20±1.96<br>[0.09 ± 0.11]	0.90±1.63<br>[0.03 ± 0.06]	U	U
C:<br>Non-<br>vaccinated<br>control	C1	U	U	3.18<br>[0.03]	U	U	U
	C2	U	3.75<br>[0.11]	3.13<br>[0.03]	U	U	†
	C3	4.40<br>[0.48]	4.45<br>[0.55]	4.69<br>[0.94]	4.18<br>[0.30]	3.42<br>[0.05]	U
	C4	3.56<br>[0.07]	U	U	U	U	U
	C5	3.39<br>[0.05]	4.33<br>[0.42]	3.77<br>[0.11]	U	3.43<br>[0.05]	U
	C6	4.01<br>[0.20]	U	3.63<br>[0.08]	U	U	U
	C7	U	3.51<br>[0.06]	3.80<br>[0.12]	U	U	U
	C8	U	U	U	U	U	U
	C9	3.45<br>[0.05]	3.77<br>[0.11]	3.83<br>[0.13]	U	U	U
	C10	3.68<br>[0.09]	3.56<br>[0.07]	U	U	U	U
	C11	4.07<br>[0.23]	4.37<br>[0.46]	3.46<br>[0.06]	U	U	U
	C12	U	4.75<br>[1.08]	3.72<br>[0.10]	3.18<br>[0.03]	U	U
	Mean	2.21±1.97<br>[0.1. ± 0.14]	2.71±2.03<br>[0.24 ± 0.33]	2.77±1.71<br>[0.13 ± 0.26]	0.61±1.45<br>[0.03 ± 0.09]	0.57±1.33<br>[0.01 ± 0.02]	U

U-undetected/ below the limit of detection of 1000 viral copies; † chicken was euthanized 16 days post challenge for humane reasons unrelated to viral challenge
